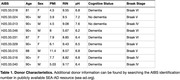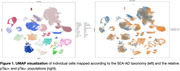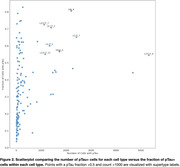# pTau‐associated gene expression signatures in the middle temporal gyrus

**DOI:** 10.1002/alz.085234

**Published:** 2025-01-03

**Authors:** Victoria M Rachleff, Joseph T Mahoney, Kyle J Travaglini, Mariano I Gabitto, Rebecca D Hodge, C Dirk Keene, Ed S Lein

**Affiliations:** ^1^ University of Washington, Seattle, WA USA; ^2^ Allen Institute for Brain Science, Seattle, WA USA; ^3^ University of Washington, School of Medicine, Seattle, WA USA; ^4^ Department of Laboratory Medicine and Pathology, University of Washington, Seattle, WA USA

## Abstract

**Background:**

Applying single‐cell RNA sequencing (scRNA‐seq) to the study of neurodegenerative disease has propelled the field towards a more refined cellular understanding of Alzheimer’s disease (AD); however, directly linking protein pathology to transcriptomic changes has not been possible at scale. Recently, a high‐throughput method was developed to generate high‐quality scRNA‐seq data while retaining cytoplasmic proteins. Tau is a cytoplasmic protein and when hyperphosphorylated is integrally involved in AD progression. The relationship between pTau accumulation and the molecular changes underlying cell type specific selective vulnerability remains poorly understood and has not been assessed in the Middle Temporal Gyrus (MTG), a critical transition zone in AD neuropathologic progression.

**Method:**

Human brain tissue was collected at rapid autopsy (postmortem interval (PMI) <12hrs). One hemisphere was embedded in alginate for fresh coronal slicing (4mm). Slabs were frozen in a dry ice isopentane slurry. Sampled MTG was processed by mechanical dissociation using a Potter‐Elvehjem tissue grinder without enzymes or detergents, followed by iodixanol gradient centrifugation, and immunolabeling (AT8/pTau and MAP2/neurons) for FACS. For each donor, MAP2+/pTau+ and MAP2+/pTau‐ fractions were collected and sequenced using the 10x Genomics Chromium Single Cell Assay. Analysis was performed using standard pipelines in R (Seurat) and Python.

**Results:**

Nine donors from Seattle Alzheimer’s Disease Brain Cell Atlas (SEA‐AD) were selected for advanced pTau pathologic distribution without end stage neurodegeneration by including Braak Stages IV‐VI (Table 1). After QC and filtering, 125,505 cells were confidently mapped to the SEA‐AD taxonomy with approximately half being pTau+ (Figure 1). While the pTau+ population primarily consisted of supragranular excitatory neurons (L2/3_IT), several less abundant supertypes also had large fractions of pTau+ cells (Figure 2). Differential expression analysis within L2/3_IT identified several upregulated AD‐related genes such as NEFM, NPTXR, and NAP1L5 in the pTau+ population.

**Conclusion:**

Disentangling the relationship between cellular vulnerability and pathology is critical to understand early AD pathogenesis. By adopting a standardized cell taxonomy and integrating datasets, we refined the definition of vulnerable cells to distinguish highly resolved pTau‐prone cell types and identified a pTau‐specific gene expression signature in the MTG.